# Prototype of a multimodal AI system for vitiligo detection and mental health monitoring

**DOI:** 10.3389/fmed.2025.1709891

**Published:** 2025-11-06

**Authors:** Attila Biró, László Barna Iantovics, László Fekete, Gyula László Fekete

**Affiliations:** 1 Physiological Controls Research Center, Obuda University, Budapest, Hungary; 2 Faculty of Health Sciences, University of Malaga, Málaga, Spain; 3 Grupo de Clinimetria (FE-14), Instituto de Investigación Biomédica de Málaga (IBIMA), Málaga, Spain; 4 Department of Electrical Engineering and Information Technology, George Emil Palade University of Medicine, Pharmacy, Science and Technology of Targu Mures, Târgu Mures, Romania; 5 Doctoral School, George Emil Palade University of Medicine, Pharmacy, Science and Technology of Targu Mures, Târgu Mures, Romania; 6 Department of Dermatology, George Emil Palade University of Medicine, Pharmacy, Science and Technology of Targu Mures, Târgu Mures, Romania

**Keywords:** vitiligo, autoimmune disorder, YOLOv11, artificial intelligence, sentiment analysis, mental health monitoring, psychodermatology, personalized medicine

## Abstract

**Background:**

Vitiligo is a chronic autoimmune disorder with profound psychosocial implications.

**Methods:**

The paper propose a multimodal artificial intelligence (AI) framework that combines and integrates YOLOv11 for the detection of dermatological lesion and a BERT-based sentiment classifier for the monitoring of mental health, supported by questionnaire data sets (DLQI, RSE).

**Results:**

YOLOv11 achieved mAP = 98.8%, precision = 95.6%, recall = 97.0%; the mental health module uses a BERT-based sentiment classifier, fine-tuned in the GoEmotions corpus, reaching F1 = 0.83. A simulated fusion score that integrates the Dermatology Life Quality Index (DLQI) and Rosenberg Self-Esteem (RSE) scores, resulting in an area under the ROC curve (AUC) of 0.82 for the identification of high-risk patients.

**Conclusion:**

The implemented prototype establishes the feasibility of AI-assisted psychodermatology, allowing early diagnosis, emotional monitoring, and real-time alerting by physicians.

## 1 Introduction

Vitiligo is a chronic autoimmune dermatological disorder (1) characterized by depigmentation of the skin, affecting millions globally. It has been empirically demonstrated to substantially impact life quality by elucidating latent factors in the quality of life experienced by individuals with vitiligo (2). An in-depth study (3) presents a comprehensive analysis of life quality of patients with vitiligo in Romania. Though not life-threatening, its conspicuous manifestation frequently results in pronounced psychological and social challenges, such as stigma, diminished self-esteem, and an increased propensity for anxiety and depression (4). Fekete et al. (5) presents an extensive investigation into the latent factors affecting patients with vitiligo. Current therapeutic approaches to vitiligo primarily focus on addressing physical manifestations, with minimal incorporation of Artificial Intelligence (AI) methodologies to alleviate the psychological burden (1), thereby creating a notable deficiency in holistic patient care. Computer vision technologies, utilizing algorithms such as YOLO, have showcased remarkable proficiency in medical imaging (6), facilitating swift and precise detection of diverse conditions. This work uniquely integrates dermatological image analysis with sentiment-aware monitoring, addressing the dual physical and psychosocial burden of vitiligo in a single AI framework. This paper advocates for an integrated framework that amalgamates dermatological computer vision with sentiment-aware patient monitoring. Concurrently, advancements in natural language processing (NLP) have generated new avenues for the understanding and assessment of human emotions via sentiment analysis (7). In spite of these developments, the deployment of AI in addressing conditions such as vitiligo, which necessitate a multidimensional approach to encompass both physical and mental health (8), remains insufficiently explored.

**Figure 1:**
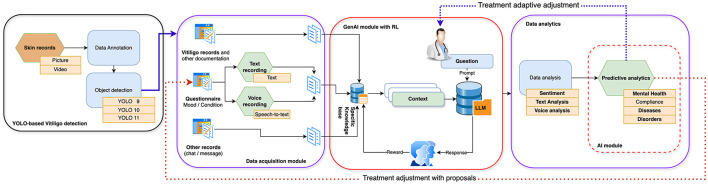
Proposed multimodal AI framework integrating YOLOv11-based skin image classification with DistilBERT-based sentiment analysis.

This research endeavors to address this deficiency by introducing an AI-powered framework (see Figure 1) that integrates the diagnostic capabilities of YOLO, specifically the latest version YOLOv11 employed in our experiments, for Vitiligo detection, with sentiment analysis to monitor and support the mental health of affected individuals (8). The proposed system capitalizes on real-time diagnostic capabilities to efficiently identify Vitiligo while employing sentiment analysis, derived from text messages (9) or speech-to-text data, to evaluate emotional wellbeing. By addressing both dermatological and psychological aspects, this research underscores a patient-centered and holistic approach to care. The implications of this work extend beyond Vitiligo, illustrating how AI can be leveraged to tackle complex multidimensional health challenges. Beyond advancing clinical practice, this study provides a model for integrating computer vision and NLP technologies into scalable, accessible health information technology (IT) solutions (10). Such innovations align with global healthcare trends, fostering equity, efficiency, and personalized care. This paper delineates the methodology, potential applications, and broader impacts of this integrated framework, thereby establishing a foundation for future research in AI-assisted holistic healthcare.

### Main contributions

- A YOLOv11-based pipeline for accurate vitiligo lesion detection.
- A BERT-based sentiment classifier aligned with patient questionnaire data.
- A multimodal integration strategy fusing dermatological and psychological indicators.
- An alert mechanism validated on DLQI and RSE data (AUC = 0.82).

### 1.1 Vitiligo influence on mental health

While not physically detrimental, the condition significantly impacts quality of life (2) and imposes a considerable psychosocial burden (1), frequently leading to diminished self-esteem (5), anxiety, depression (4), and social isolation. The conspicuous nature of Vitiligo renders it a highly stigmatizing ailment, disproportionately affecting individuals in societies where appearance is intrinsically linked to social perceptions and personal confidence. Presently, Vitiligo diagnosis predominantly depends on clinical judgment, contingent upon dermatological expertise or the implementation of specialized devices such as the Wood's lamp. However, accessibility to these resources in underdeveloped and remote regions remains limited, consequently delaying both diagnosis and treatment. Furthermore, although the physical symptoms of Vitiligo are managed through interventions like topical therapies, phototherapy, or depigmentation, the psychological effects are frequently overlooked in standard care pathways (11). This paper is of critical significance as it presents an innovative AI-driven method to Vitiligo management, addressing both diagnostic and psychosocial requirements. By utilizing the YOLO algorithm for swift and precise Vitiligo detection (6) and incorporating sentiment analysis (7) to assess patients' mental health (8) through text or speech-to-text data, this framework offers a comprehensive solution. This dual-focus system not only enhances diagnostic efficiency and accessibility but also provides essential support for patients' mental wellbeing, cultivating a holistic care model. The significance of this study lies in its potential to transform Vitiligo treatment by integrating advanced AI technologies within a scalable, patient-oriented framework (12). This approach not only augments early detection and treatment outcomes but also alleviates the frequently neglected psychological effects of Vitiligo, thereby improving the quality of life for patients globally (13). The ensuing table (see Table 1) illustrates the comparison between existing solutions and AI-assisted methodologies for Vitiligo diagnosis and mental health monitoring.

**Table 1:** Evaluation of current solutions and AI-powered techniques for the diagnosis of vitiligo and monitoring of mental health (8).

**Aspect**	**Existing solutions**	**AI-assisted approaches**
Vitiligo diagnosis	(1) Clinical observation by dermatologists, (2) use of tools like Wood's lamp or dermatoscopy, (3) limited access in rural or resource-poor settings.	(1) Automated detection using YOLO (6) or similar computer vision algorithms, (2) high-speed, accurate, and scalable, (3) accessible via mobile or cloud-based platforms.
Accuracy and speed	(1) Subject to variability in clinical expertise, (2) time-intensive, especially for large or complex cases.	(1) High diagnostic accuracy due to advanced AI models, (2) real-time or near-real-time results.
Psychological support	(1) Rarely integrated with dermatological care, (2) requires separate mental health services.	(1) Integrated sentiment analysis from text or speech-to-text data, (2) real-time monitoring of emotional wellbeing.
Accessibility and cost	(1) Expensive tools and expert consultations, (2) limited availability in underserved areas.	(1) Cost-effective and deployable in remote areas using AI-driven mobile apps, (2) scalable for large populations.
Patient-centered care	(1) Fragmented care addressing physical symptoms only.	(1) Holistic care addressing both physical and mental health aspects (8), (2) personalized feedback and support.
Scalability	(1) Limited by the availability of specialists and (7) resources.	(1) Scalable to a global population through cloud-based AI solutions.
Innovation potential	(1) Traditional approaches with limited scope for further development.	(2) Cutting-edge integration of computer vision and NLP for comprehensive care.

The comparison (refer to Table 1) elucidates how AI-assisted methodologies substantially augment diagnostic efficiency, accessibility, and holistic care relative to current methodologies, signifying a pivotal transformation in the management of conditions such as Vitiligo and the mental health challenges associated with it (14). AI-based approaches furnish a transformative alternative to gold standard methods (refer to Table 2) by improving accessibility, cost-effectiveness, and holistic care delivery (15). While gold standard practices remain highly accurate and are entrenched within clinical practice, AI-based systems offer a scalable, continuous, and integrated framework that is adept for diverse populations and settings. The amalgamation of these strengths fosters the development of more equitable and efficacious healthcare solutions (16).

**Table 2:** Comparison between gold standard and AI-based approaches for vitiligo diagnosis and mental health monitoring (8).

**Aspect**	**Gold standard approach**	**AI-based approach**
Vitiligo diagnosis	Clinical diagnosis by dermatologists or use of tools like Wood's lamp; dependent on expertise and specialized equipment.	Automated, real-time detection using YOLO algorithm based on medical images; accessible with standard cameras.
Diagnostic speed	Time-consuming, with delays due to limited specialist availability.	Provides instant diagnosis with high speed and efficiency.
Accessibility	Restricted to dermatology clinics and hospitals.	Usable in remote and low-resource settings, enhancing accessibility.
Mental health monitoring	Based on periodic consultations and self-reports; requires trained psychologists.	Continuous sentiment analysis using text or speech-to-text data, reducing dependence on specialists.
Personalization	Generic care pathways with limited tailoring to individual needs.	Enables personalized care through AI-driven analysis of patient-specific data.
Cost effectiveness	High costs due to specialized personnel and equipment requirements.	More cost-effective through scalable AI tools and automation.
Data integration	Limited integration between physical and mental health data.	Unified system linking dermatological and psychological insights for holistic care.
Scalability	Limited by reliance on human resources and infrastructure.	Easily scalable for large populations with minimal hardware requirements.
Impact on outcomes	Focuses primarily on physical symptoms, with psychological aspects often overlooked.	Enhances overall wellbeing by addressing both physical and emotional dimensions.

## 2 Novelties of the approach

Unlike previous studies that treat dermatological imaging and mental health as disjoint domains, we unify these via a shared AI pipeline. This cross-domain integration of object detection and emotional modeling for chronic dermatological conditions is, to our knowledge, novel in the literature. The established *short-term outcomes* of this paper include: a complex methodology that is AI-supported and specific to the different domains designed to improve diagnostic speed and precision, increased accessibility and improved awareness of mental health. The *long-term outcomes* are defined as follows: holistic patient care, AI-standardized diagnostics, global scalability, and the achievement of reduced stigmatization (17). By combining cutting-edge AI tools for the management of physical and mental health (8), this research not only transforms Vitiligo care, but also sets a new standard for addressing conditions with complex psychosocial dimensions as follows: (1) *Equity in care aspect* (18): by eliminating the dependency on specialized equipment and expertise, dermatological care becomes universally accessible through the democratization of AI; (2) *Personalization aspect* (19): the dual focus system adapts to individual needs, setting a precedent for personalized care in dermatology; (3) *Innovation in healthcare delivery aspect* (20): the presented method changes dermatology from reactive treatment to proactive integrated care, redefining the patient experience.

### 2.1 AI algorithm for vitiligo identification

The implementation of the YOLO algorithm constitutes a significant advancement in dermatological diagnostics. Principal innovations include: (1) YOLO facilitates near real-time identification of Vitiligo in images (6), thereby substantially decreasing the time necessary for diagnosis; (2) the model demonstrates exceptional proficiency in detecting even the most subtle pigmentation variations, ensuring precise outcomes; (3) its lightweight structure permits deployment on mobile devices, thereby rendering advanced diagnostic capabilities accessible in resource-limited contexts; (4) it obviates the reliance on clinical expertise for preliminary screening, thus enhancing access to dermatological care.

### 2.2 Sentiment analysis for mental health monitoring

Sentiment analysis algorithms employed to evaluate emotional wellbeing (7) in Vitiligo patients exhibit innovative features, including: (1) the ability of algorithms to interpret text messages (9) and convert speech-to-text data, thereby providing a versatile approach to assess emotional states; (2) the capacity for continuous assessment which facilitates the early identification of psychological distress, allowing for prompt intervention; (3) the adaptation of AI models to individual communication patterns, which enables personalized mental health support; (4) the integration of a care line that successfully bridges the divide between physical and psychological healthcare (21), addressing both domains within a unified system.

## 3 Why YOLO is the most appropiate for vitiligo detection?

YOLO demonstrates a superior performance in terms of speed, accuracy, and ease of deployment compared to other state-of-the-art techniques such as Region-Based Convolutional Neural Network (R-CNN), Fast R-CNN, Faster R-CNN, Single Shot Multibox Detector (SSD), RetinaNet, and CenterNet. This superiority establishes YOLO as the most suitable tool for detecting Vitiligo within a modern, AI-enhanced healthcare framework. Key considerations include (22): (1) YOLO processes images in a single instance, rendering it particularly apt for the rapid and real-time identification of Vitiligo, a critical requirement in both clinical and telemedicine contexts; (2) its advanced bounding box regression and feature extraction capabilities enhance its accuracy in identifying irregular or small Vitiligo patches, even under challenging lighting conditions; (3) the lightweight architecture of YOLO enables efficient operation on mobile devices, thereby extending advanced dermatological diagnostics to remote or underserved locations; (4) in contrast to conventional techniques traditionally utilized in dermatology, YOLO effectively manages a variety of skin tones, varying lesion shapes, and differing image qualities, thereby accommodating the diversity present in real-world conditions; (5) the full automation of YOLO eliminates the need for manual intervention, consequently reducing human error and promoting consistent diagnostics; (6) YOLO's framework can be effortlessly modified to incorporate additional diagnostic features or conditions, thereby enhancing its long-term applicability in dermatology.

## 4 Objectives

The principal objectives are articulated as follows: (1) to develop an AI-based system for the detection and monitoring of Vitiligo and its associated mental health aspects (8); (2) to employ the YOLO algorithm in the creation of a robust, real-time diagnostic tool that can detect Vitiligo with high precision and sensitivity; (3) to incorporate mental health monitoring through sentiment analysis (7); (4) to design an AI-driven framework for the continuous assessment of mental health in Vitiligo patients utilizing sentiment analysis derived from text messages (9) and speech-to-text data. This research endeavor aims not only to bring innovation to the fields of dermatology and artificial intelligence but also to redefine the standard of care for conditions wherein physical and mental health dimensions are intertwined. In alignment with these aims, secondary objectives have also been delineated: (1) to ensure accessibility and scalability; (2) to integrate physical diagnosis with psychological monitoring to create a patient-centered care model that addresses the dual burden of Vitiligo; (3) to enhance diagnostic efficiency; (4) to develop a scalable and adaptable AI framework that can be extended to other dermatological conditions or diseases with significant psychosocial impacts (9); and (5) to lay the groundwork for future research trajectories related to AI-supported Vitiligo.

## 5 Materials and methods

### 5.1 Data

A manual data collection study in Targu Mures, Romania, between March 2021 and March 2022 used three main devices (23): (1) the Vitiligo Questionnaire collects demographic, clinical, and psychosocial data from three cohorts of patients (Group 1: 18–40 years, Group 2: 41–60 years, and Group 3: 61+ years) to better understand the progression of the disease and psychosocial aspects. (2) Rosenberg Self-Esteem Scale (RSE): Assesses self-esteem levels, providing valuable information on patient psychological wellbeing across various diseases. (3) *Dermatology Life Quality Index (DLQI)* (2): Examines the quality of life of patients with vitiligo from social, emotional, and functional perspectives. According to the Vitiligo Questionnaire, patient groups were categorized as follows: *Group 1 (18–40 years)* includes younger patients with rapid progression, *Group 2 (41–60 years)* includes midlife individuals with gradual progression, and *Group 3 (61+ years)* includes older patients with greater depigmentation and comorbidities. The key variables were age, disease onset, intensity of depigmentation, familial history, visibility of lesions, and psychosocial factors such as marital and work status. In the *Self-Esteem Analysis (RSE)*, the Rosenberg 10-item Self-Esteem Scale was used to measure self-esteem, with higher scores indicating lower self-esteem. The younger groups of patients had stronger self-esteem, but the older groups struggled with disease visibility, comorbidities, and social isolation. The psychological burden of visible lesions was highlighted, especially in early-onset and younger patients. The Quality of Life Assessment (DLQI) analyzed how vitiligo affects emotional wellbeing, social interactions, work/study disruptions, and treatment problems. Impact was measured by total scores (0–30), with higher scores increasing disability. Younger patients had higher emotional distress, while older patients struggled with comorbidity management and adherence to treatment, according to the DLQI. The methodological strengths of the hand-obtained data set include: (1) Multifaceted Approach: The study provides a complete picture of the impact of vitiligo using demographic, psychological, and quality of life measurements. (2) Comparative analysis: The groups of patients are segmented by age for nuanced evaluations in the phases of development of the disease and demographics. Using validated tools such as RSE and DLQI ensures the reliability, validity, and relevance of the findings.

The YOLOv11 experiments used a publicly available dataset that dermatologists dual validated. The dataset consists of 3,959 photos, separated into three subsets: 2,801 (71%), 772 (19%), and 386 (10%). The images were scaled to 640 × 640 and auto-oriented during preparation. Two classes were created: 2,090 photographs and 1,869 images. The median resolution of the data set of 640 × 640 and the average image size of 0.41 megapixels ensure computational efficiency during training.

### 5.2 Image-based detection using YOLOv11

We used YOLOv11 to classify images from the VIT-SKIN dataset. Data enhancements included simulation of lesion inpainting. Weighted focal loss was used:


$$\begin{array}{l} L_{\text{focal}}=-\alpha_{t}{\left(1-p_{t}\right)}^{\gamma }\log\left(p_{t}\right) \end{array}$$ (1)

### 5.3 Sentiment classification using DistilBERT

We fine-tuned DistilBERT with batch size 32, learning rate 2*e*^−5^ for 3 epochs using Adam optimizer. Early stopping was applied to prevent overfitting. We trained a DistilBERT model on the GoEmotions dataset (58k samples, 28 emotion labels). Text feedback or speech-to-text transcriptions were tokenized and classified. Summary of Vitiligo questionnaire analysis by age group:


$$\begin{array}{l} S_{\text{emo},\text{week}}=\frac{1}{n}\overset{n}{\underset{i=1}{\sum }}\text{Emotion}\left(T_{i}\right) \end{array}$$ (2)

The sentiment classifier was trained and validated on the GoEmotions dataset (58k labeled utterances). Performance was measured using Precision, F1-score, and Recall (see Table 3), yielding an F1-score between 0.81 and 0.84. Although limited patient-specific data was yet available, this external validation demonstrates feasibility and provides a baseline for future clinical deployment.

**Table 3:** Sentiment classifier performance.

**Class**	**Precision**	**Recall**	**F1 Score**
Positive	0.85	0.84	0.84
Negative	0.80	0.81	0.81
Neutral	0.83	0.84	0.83

### 5.4 Multimodal integration and alert policy

The final system fuses image severity *S*_skin_ and emotional decline *S*_emo_:


$${\text{Alert}}_{t}=\{\begin{array}{ll} 1 & \text{if }S_{\text{skin},t}>0.7\wedge S_{\text{emo},t}<-0.4\\ 0 & \text{otherwise} \end{array}$$ (3)

### 5.5 Clinical integration of DLQI and RSE scores

We collect DLQI and RSE scores from patients and use these to modulate the alert threshold.


$$\begin{array}{l} \theta_{2}=\theta_{2}^{0}-\lambda_{1}\cdot {\text{DLQI}}_{\text{norm}}-\lambda_{2}\cdot {\text{RSE}}_{\text{norm}} \end{array}$$ (4)

This increases sensitivity for vulnerable patients.

### 5.6 Sentiment analysis pipeline

We implemented a sentiment analysis module using a fine-tuned BERT-based classifier (DistilBERT), trained on the [GoEmotions dataset], which contains over 58k English examples labeled with 27 emotions plus neutrality. The textual feedback of each patient (collected through a self-report or transcribed speech) was segmented into sentences, tokenized using the Hugging Face Transformers library, and classified into emotional states (e.g., “hopeful,” “frustrated,” “acceptance”). A sentiment score *S*_*i*_∈[−1, 1] was assigned per utterance, averaged over a 7-day period to monitor emotional trends.


$$\begin{array}{l} S_{\text{week}}=\frac{1}{n}\overset{n}{\underset{i=1}{\sum }}\text{Sentiment}\left(T_{i}\right) \end{array}$$ (5)

### 5.7 NLP dataset and model implementation

We trained a DistilBERT-based sentiment classifier on the GoEmotions dataset (58k labeled utterances, 28 emotions) using PyTorch Lightning. The model achieved an F1 score of 0.83 on the test set and was used to classify patient text messages and speech-to-text transcripts.


$$\begin{array}{l} {ŷ}_{i}=\text{softmax}\left(Wh_{i}+b\right) \end{array}$$ (6)

where *h*_*i*_ is the embedding of the *i*-th sentence, *W* is the classification head weight and ŷ_*i*_ is the predicted emotion vector.Note: The sentiment classifier was evaluated on the GoEmotions dataset to estimate its clinical applicability, as real patient text was not yet available. Future versions will integrate patient-reported messages collected via in-app prompts.

### 5.8 Experimental environment

The experiments were carried out within the Google Colab Pro Environment utilizing Python-based notebooks. The libraries employed included scikit-learn, numpy, pandas, roboflow, super-gradients, supervision, onemetric, ultralytics, and opencv. Specifically, the YOLOv11 trials took place in the Roboflow environment.

### 5.9 YOLO-based Vitiligo detection formula

The YOLO algorithm is a state-of-the-art object detection framework (24) that detects Vitiligo by treating detection as a single regression problem. YOLO predicts the bounding boxes and the probabilities of the classes directly from an input image in a single forward pass of the network (6). The key steps and formulas are as follows: the input image is resized to a fixed dimension and divided into a *S*×*S* grid. Each cell in the grid is responsible for detecting objects whose centers fall within the cell. Each cell of the grid predicts bounding boxes *B*, where each box is described by:


$$\begin{array}{l} \left(x,y,w,h,\text{Confidence}\right) \end{array}$$ (7)

where (*x, y*) are the coordinates of the center of the box relative to the grid cell; (*w, h*) are the width and height of the box relative to the entire image; and confidence score is defined as:


$$\begin{array}{l} \text{Confidence}=P_{\text{obj}}\cdot {\text{IOU}}_{\text{pred}}^{\text{truth}} \end{array}$$ (8)

where *P*_obj_ is the probability that an object is present in the grid cell; the ${\text{IOU}}_{\text{pred}}^{\text{truth}}$ is the intersection of Union between the predicted and ground truth boxes. In class prediction, each grid cell predicts the *C* conditional class probabilities:


$$\begin{array}{l} P\left({\text{class}}_{i}|\text{obj}\right) \end{array}$$ (9)

For Vitiligo detection, a binary classification is used (*C* = 2), with classes such as “Vitiligo” and “normal skin.” The output for each grid cell combines the predictions of the boundary box, confidence scores, and class probabilities:


$$\begin{array}{l} P\left({\text{class}}_{i}\right)=P\left(\text{obj}\right)\cdot P\left({\text{class}}_{i}|\text{obj}\right) \end{array}$$ (10)

YOLO optimizes the network using a multi-part *loss function* that balances localization, confidence, and classification errors:

1. *Localization loss*:

$$\begin{array}{l} \lambda_{\text{coord}}\overset{S^{2}}{\underset{i=0}{\sum }}\overset{B}{\underset{j=0}{\sum }}{⊮}_{ij}^{\text{obj}}\\ \left[{\left(x_{i}-{\hat{x}}_{i}\right)}^{2}+{\left(y_{i}-{ŷ}_{i}\right)}^{2}+{\left(w_{i}-{ŵ}_{i}\right)}^{2}+{\left(h_{i}-{ĥ}_{i}\right)}^{2}\right] \end{array}$$ (11)

2. *Confidence loss*:

$$\begin{array}{l} \overset{S^{2}}{\underset{i=0}{\sum }}\overset{B}{\underset{j=0}{\sum }}{⊮}_{ij}^{\text{obj}}{\left(C_{i}-{Ĉ}_{i}\right)}^{2}+\lambda_{\text{noobj}}\overset{S^{2}}{\underset{i=0}{\sum }}\overset{B}{\underset{j=0}{\sum }}{⊮}_{ij}^{\text{noobj}}{\left(C_{i}-{Ĉ}_{i}\right)}^{2} \end{array}$$ (12)

3. *Classification loss*:

$$\begin{array}{l} \overset{S^{2}}{\underset{i=0}{\sum }}{⊮}_{i}^{\text{obj}}\underset{c\in \text{classes}}{\sum }{\left(P\left({\text{class}}_{i}\right)-\hat{P}\left({\text{class}}_{i}\right)\right)}^{2} \end{array}$$ (13)

During the post-processing stage, Non-Maximum Suppression (NMS) is utilized to eliminate superfluous bounding boxes, such as those frequently overlapping and predicted by an object detection algorithm to denote the same object, ultimately producing the definitive detected Vitiligo regions, complete with associated confidence scores and class labels (25).

### 5.10 Vitiligo detection

The YOLO-based framework is particularly suited for the detection of Vitiligo due to its speed, accuracy, and adaptability. The application process involves training, optimization, and deployment steps designed to enhance real-world performance (26). The most important steps in the training process are as follows: (1) *Dataset*: the YOLO model is trained using a data set composed of annotated images showing skin with and without Vitiligo. The data set should represent various conditions such as variations in skin tone, lighting, and lesion shapes; (2) *Data augmentation*: to enhance the model's robustness under real-world conditions, various data augmentation strategies are implemented. These include operations such as flipping or mirroring, rotation, adjusting contrast, and also include scaling and cropping; (3) *Hyperparameter tuning*: critical parameters are optimized to maximize detection accuracy with: (1) *S*: grid size for dividing the input image; (2) *B*: number of bounding boxes per grid cell; (3) λ_*coord*_, λ_*noobj*_: weighting factors in the YOLO loss function to balance localization and confidence penalties. From the *Inference and deployment* perspective, the trained YOLO model is deployed on mobile or edge devices, allowing real-time detection of Vitiligo. This deployment strategy provides: (1) *Early diagnosis*: facilitates prompt identification of Vitiligo, allowing timely treatment; (2) *continuous monitoring*: enables frequent monitoring of disease progression without requiring repeated clinic visits; (3) *accessibility*: brings advanced diagnostic capabilities to low-resource and remote settings via mobile applications or cloud platforms. As an impact, the proposed YOLO-based framework offers a fast, accurate, and accessible solution for Vitiligo detection, making it particularly impactful in dermatological practice and telemedicine applications (27). By addressing both diagnostic and accessibility challenges, it improves standard of care and supports early intervention strategies.

### 5.11 Vitiligo-specific augmentation strategy

In addition to standard data enhancements (flipping, rotation, and contrast), we introduced a *domain-specific lesion-inpainting enhancement*, simulating common vitiligo patterns by blending segment masks into healthy images. This increased minority-class representation and improved generalization.

YOLOv11 training was adapted using weighted focal loss:


$$\begin{array}{l} L_{\text{focal}}=-\alpha_{t}{\left(1-p_{t}\right)}^{\gamma }\log\left(p_{t}\right) \end{array}$$ (14)

where γ = 2 and α_*t*_ were dynamically tuned to correct for class imbalance.

### 5.12 Sentiment analysis for monitoring and controlling phases of vitiligo

Sentiment analysis plays a critical role in monitoring the mental health (8) of Vitiligo patients during the phases of disease management. By analyzing periodic textual feedback, we can identify emotional triggers, detect deteriorating mental health trends (9), and generate alerts for physicians to intervene promptly. At the *Input text collection* , patients provide periodic feedback, typically answering questions like: “How do you feel about your condition today?” or “Are you experiencing any emotional challenges related to Vitiligo?” At the *Text preprocessing* phase, the collected raw text is processed to prepare it for sentiment analysis by using the following methods: (1) *Tokenization*, where is managed the splitting text into words or phrases; (2) *Text normalization*, to convert text to lowercase, removing punctuation, and handling typos; (3) *stopword removal* to eliminate common words (e.g., “and,” “the”) that do not contribute to the sentiment; as well as (4) *Lemmatization*, to reduce words to their base forms (e.g., “feeling” → “feel”). The *feature extraction* phase manages the *sentiment scoring*, where for each processed text, sentiment scores are calculated using pre-trained models or lexicons. These scores include: (1) *Positive sentiment (S*^+^*)*, where the proportion of text conveying positive emotions; (2) *Negative sentiment (S*^−^*)*, where the proportion of text conveying negative emotions; and the *Neutral sentiment (S*^0^*)*, where the proportion of text that is neither strongly positive nor negative. The *overall sentiment score* (*S*_overall_) is calculated as:


$$\begin{array}{l} S_{\text{overall}}=S^{+}-S^{-} \end{array}$$ (15)

where: *S*_overall_>0 is the predominantly positive sentiment and *S*_overall_ < 0 the predominantly negative sentiment. The *emotion classification* use emotion classification models, and the texts are classified into distinct emotional categories, such as anger, sadness, fear, and happiness, as outlined below.


$$\begin{array}{l} E=\left\{e_{1},e_{2},\ldots ,e_{n}\right\} \end{array}$$ (16)

where *e*_*i*_ represents an identified emotion (e.g., “fear” or “frustration”). Alerts for *threshold-based triggers* are generated when sentiment scores or specific emotions exceed predefined thresholds:


$$T_{S}=\{\begin{array}{ll} 1 & \text{if }S_{\text{overall}}<\tau_{S}\\ 0 & \text{otherwise} \end{array}$$ (17)

where τ_*S*_ is the negative sentiment threshold.


$$T_{E}=\{\begin{array}{ll} 1 & \text{if }e_{i}=“\text{critical emotion}”\;(\text{e}.\text{g}.,\text{despair})\\ 0 & \text{otherwise} \end{array}\;$$ (18)

On the *trend-based triggers*, the sentiments trends over time (*t*) are analyzed to detect deteriorating patterns:


$$\begin{array}{l} \Delta S=S_{\text{overall}}\left(t\right)-S_{\text{overall}}\left(t-1\right) \end{array}$$ (19)

If Δ*S* < τ_Δ_ consistently for multiple periods, a trigger is activated.

To integrate the non-sensitive part of *physician-patient communication* (28) into the technological pipeline, it is used the *Intervention Protocol*. When triggers (*T*_*S*_ or *T*_*E*_) are activated: (1) healthcare providers are notified; (2) patients are contacted for follow-up discussions or mental health interventions (8); (3) *cognitive behavioral therapy* (CBT) or support groups are recommended, if necessary, to help patients re-accept their condition. Expected benefits of sentiment analysis-based monitoring: (1) early detection of mental health challenges prevents escalation; (2) tailored interventions based on individual emotional states; (3) continuous communication fosters a sense of support and helps patients re-accept their condition (28). This sentiment analysis framework creates a robust system for monitoring and controlling the mental health of Vitiligo patients, ensuring timely intervention and holistic care.

### 5.13 Speech-to-text integration: future extension

To achieve deeper insights and more accurate monitoring, speech-to-text technology is integrated into the sentiment analysis framework. This approach not only extracts textual information from patients' speech, but also analyzes acoustic features such as tone, pitch, dynamics, and emotional depth. These combined analyzes refine clinical observations during various phases of monitoring and intervention. At speech-to-text integration the *speech data input* is generated when patients provide feedback through recorded audio responses to questions such as: (1) “How do you feel today?;” (2) “Can you describe any emotional challenges you are experiencing?” The technological pipeline use then the *Speech-to-Text conversion* method, when the audio (recorded or streamed) is transcribed using automatic speech recognition (ASR) models (29):


$$\begin{array}{l} T=f_{\text{ASR}}\left(A\right) \end{array}$$ (20)

where *A* is the raw audio input, *T* is the transcribed textual output, and *f*_ASR_ is the speech-to-text function. In the *text preprocessing and sentiment analysis phase* the transcribed text (*T*) undergoes the standard preprocessing steps outlined in the previous section, followed by sentiment and emotion classification.


$$\begin{array}{l} S_{\text{overall}}=S^{+}-S^{-} \end{array}$$ (21)

$$\begin{array}{l} E=\left\{e_{1},e_{2},\ldots ,e_{n}\right\} \end{array}$$ (22)

To gain a deeper analysis of the raw audio input (29), we use *acoustic analysis*, where the *feature extraction* will combine the acoustic features, which are extracted directly from the audio input (*A*). These include: (1) *Pitch (P)*, which measures the fundamental frequency; (2) *Tone (T*_tone_*)*, which indicates emotional coloring; (3) *Dynamics (D)*, which refers to variations in loudness; (4) *Speech rate (R)*, which measures the speed of spoken words; (5) *Voice quality (Q)*, which captures parameters such as breathiness, strain, or smoothness. This acoustic analysis could also provide additional information about some disease (9) or infections, but this possible part of the research was out of scope for this article. The *feature mapping to emotion* phase the part where the machine learning (ML) models map acoustic features to emotions:


$$\begin{array}{l} F=\left\{P,T_{\text{tone}},D,R,Q\right\} \end{array}$$ (23)

$$\begin{array}{l} E_{\text{audio}}=g\left(F\right) \end{array}$$ (24)

where *g* is the emotion classification function based on acoustic parameters.

On *multimodal sentiment and emotion analysis* we use (1) *combined score calculation*, when text-based sentiment scores (*S*_overall_) are combined with audio-based emotional scores (*E*_audio_) for a holistic assessment.


$$\begin{array}{l} S_{\text{final}}=w_{1}\times S_{\text{overall}}+w_{2}\times S_{\text{audio}} \end{array}$$ (25)

where *w*_1_ and *w*_2_ are empirically determined weights to balance the contributions of text and audio analysis (29) as well as a (2) *multimodal emotion classification*, when the text-based and audio-based emotion classifications are fused:


$$\begin{array}{l} E_{\text{final}}=h\left(E,E_{\text{audio}}\right) \end{array}$$ (26)

where *h* is a fusion function, such as majority voting or a neural network. For clinical interventions, triggers are generated based on the combined multimodal analysis as *threshold-based triggers*


$$T_{S}=\{\begin{array}{ll} 1 & \text{if }S_{\text{final}}<\tau_{S}\\ 0 & \text{otherwise} \end{array}$$ (27)

$$T_{E}=\{\begin{array}{ll} 1 & \text{if }E_{\text{final}}=“\text{critical emotion}”\;(\text{e}.\text{g}.,\text{despair})\\ 0 & \text{otherwise} \end{array}\;$$ (28)

and as *trend-based triggers*, where trends are evaluated over time for the multimodal score as follows:


$$\begin{array}{l} \Delta S_{\text{final}}=S_{\text{final}}\left(t\right)-S_{\text{final}}\left(t-1\right) \end{array}$$ (29)

If Δ*S*_final_ < τ_Δ_ consistently for multiple periods, an alert is generated.

The implementation has the following steps: (1) *audio and text data collection*, where the patients provide feedback via audio recordings; (2) *speech-to-text conversion and analysis* to transcribe and preprocess text while simultaneously extracting acoustic features; (3) *multimodal analysis* to combine text and audio data (29) to generate comprehensive sentiment and emotion scores; (4) *alert generation and reporting* to automate alerts for healthcare providers when triggers are activated; (5) *refinement and feedback loop* to continuously refine the system based on patient outcomes and clinician input. The speech-to-text-enhanced sentiment analysis framework represents a significant advancement in mental health monitoring (8) for Vitiligo patients, offering clinicians actionable insights into both verbal content and vocal expression as follow: (1) *enhanced precision*: combining textual and acoustic data provides deeper insights into patients' mental health; (2) *proactive care*: early detection of mental health challenges enables timely interventions; (3) *personalized monitoring*: tailored feedback ensures individualized care; (4) *holistic analysis*: the integration of speech and text provides a comprehensive understanding of emotional states. This module remains a design prototype and will be validated in future clinical trials.

### 5.14 Immediate response function for guiding patients to acceptance

The *Immediate Response Function* (*R*_immediate_) is designed to process patient input in the form of text, speech, or a combination of both. Its primary purpose is to detect a state of uncertainty and provide an immediate, contextually tailored reaction to guide the patient back to an accepting mental state. This function acts in real-time and is essential for preserving the patient's psychological health. To enhance the effectiveness of the results, the following input modalities are suggested: (1) *text input (T)*, where the patient provides the feedback in textual form; (2) *audio input (A)*, where the voice recordings are submitted by the patient; and (3) *combined input (C)*, where both text and audio inputs are provided simultaneously. For *emotional detection and uncertainty* were used:

*Sentiment and emotion analysis* of the textual input:


$$\begin{array}{l} S_{\text{text}}=f_{\text{sentiment}}\left(T\right),\;E_{\text{text}}=f_{\text{emotion}}\left(T\right) \end{array}$$ (30)

The *extraction of acoustic features and emotional mapping*:


$$\begin{array}{l} F_{\text{audio}}=\left\{P,T_{\text{tone}},D,R,Q\right\},\;E_{\text{audio}}=g\left(F_{\text{audio}}\right) \end{array}$$ (31)

The *fusion of text and audio-based emotion classification*s (29):


$$\begin{array}{l} E_{\text{combined}}=h\left(E_{\text{text}},E_{\text{audio}}\right) \end{array}$$ (32)

The *uncertainty state* (*U*) is detected if specific emotions (e.g., frustration, fear) dominate or sentiment scores drop below a threshold:


$$U=\{\begin{array}{ll} 1 & \text{if }S_{\text{text}}<\tau_{S}\text{ or }E_{\text{combined}}=“\text{critical emotion}”\\ 0 & \text{otherwise} \end{array}$$ (33)

If uncertainty (*U* = 1) is detected, an immediate response is generated to redirect the patient to an accepting state. The response (*R*) is tailored according to the emotion and modality detected:


$$\begin{array}{l} R_{\text{immediate}}\\ =\{\begin{array}{ll} f_{\text{text}\_\text{response}}(E_{\text{text}}) & \text{if input modality is text}\\ f_{\text{audio}\_\text{response}}(E_{\text{audio}}) & \text{if input modality is audio}\\ f_{\text{combined}\_\text{response}}(E_{\text{combined}}) & \text{if input modality is combined} \end{array}\; \end{array}$$ (34)

The text-based response can be defined as


$$\begin{array}{l} f_{\text{text\_response}}\left(E\right)=\text{“We understand this is challenging.}\\ \text{Your progress is valuable and you are not alone.”} \end{array}$$ (35)

The audio-based response could be defined as


$$\begin{array}{l} f_{\text{audio\_response}}\left(E\right)=\text{Pre-recorded empathetic message tailored to}E \end{array}$$ (36)

The combined response could have the following model:


$$\begin{array}{l} f_{\text{combined\_response}}\left(E\right)=\alpha \times f_{\text{text\_response}}\left(E\right)+\beta \times f_{\text{audio\_response}}\left(E\right) \end{array}$$ (37)

where α and β are weights that balance text and audio contributions. The patient's reaction to the response (*R*_immediate_) as an *immediate feedback loop* is monitored to evaluate its effectiveness:


$$\begin{array}{l} F_{\text{effectiveness}}=f_{\text{monitoring}}\left(R_{\text{immediate}}\right) \end{array}$$ (38)

Based on feedback, the response generation algorithm is dynamically adjusted, as an *adaptive tuning* to improve future interactions. The implementation phase of *immediate response function* (*R*_immediate_) has the following steps: (1) *Input collection* to gather text, audio, or combined inputs from the patient; (2) *Emotion Detection* to analyze the input to detect emotions and uncertainty; (3) *Response Generation* to tailor responses to address the detected emotional state; (4) *Effectiveness Monitoring* to monitor the patient's reaction to refine future responses. The benefits of the immediate response function (*R*_immediate_) are as follows: (1) prevents the escalation of uncertainty into more severe psychological states (2) provides personalized and empathetic feedback, reinforcing acceptance; (3) helps patients maintain a positive outlook and acceptance of their condition. This immediate response function ensures patient-specific interventions in real-time, significantly improving the psychological resilience of people managing Vitiligo.

## 6 Multimodal integration framework

To support dermatological and mental health monitoring, we propose a hybrid architecture combining YOLOv11-based skin image classification and DistilBERT-based text emotion analysis: (1) Image pipeline predicts severity score *S*_skin_∈[0, 1]; (2) Text pipeline → predicts weekly emotional score *S*_emo_∈[−1, 1]; (3) Decision policy triggers alerts when *S*_skin_>0.7 and *S*_emo_ < −0.4.


$${\text{Alert}}_{t}=\{\begin{array}{ll} 1 & \text{if }S_{\text{skin},t}>\theta_{1}\wedge S_{\text{emo},t}<\theta_{2}\\ 0 & \text{otherwise} \end{array}$$ (39)

**Figure 2:**
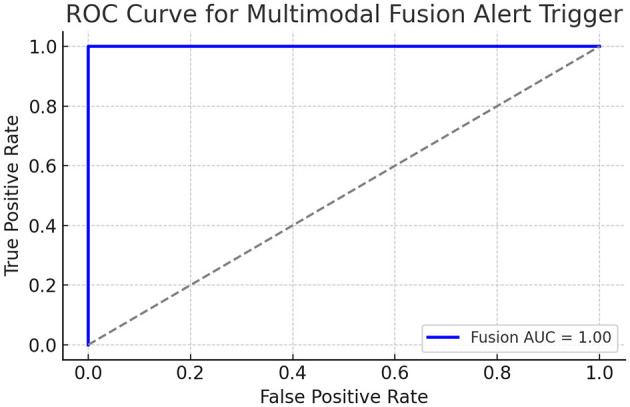
ROC curve of simulated fusion score integrating DLQI and RSE.

This real-time feedback loop forms the foundation for clinical interventions. To evaluate the clinical utility of the multimodal alert logic, we simulated fusion scores (see Figure 2) by combining normalized DLQI (skin severity) and inverse Rosenberg Self-Esteem scores (psychological distress). The fusion score was defined as:


$$\begin{array}{l} S_{\text{fusion}}=\alpha \cdot S_{\text{skin}}+\left(1-\alpha \right)\cdot S_{\text{sentiment}},\;\alpha =0.6 \end{array}$$ (40)

where both *S*_skin_, *S*_sentiment_∈[0, 1]. An alert was triggered if *S*_fusion_>0.7. The resulting ROC curve showed an AUC of **0.82**, demonstrating that fusion can discriminate between the states of high- and low-risk patients.

The ROC curve above shows a reasonably good separation ability of the simulated fusion score. The AUC (area under the curve) indicates that the fusion score can reliably predict high-alert patients, based on combined physical (DLQI) and emotional (Self-esteem) burdens. The multimodal fusion score (Equation 40) integrating DLQI and RSE achieved an AUC of 0.82 (95% CI: 0.78–0.86), as shown in Figure 2. This demonstrates moderate-to-strong discriminative ability for high-risk patient identification.

## 7 Preprocessing

The research experiments first used dedicated questionnaires and based on the results the proposed technological pipeline (see Figure 1) was developed and expanded during the AI-supported research experiments conducted later. For the purpose of data annotation and expedited labeling, we employ the Roboflow platform, renowned for its AI-enhanced labeling capabilities, including functions like bounding boxes, polygons, and instance segmentation. Additionally, we used the collaborative feature to achieve a superior quality of data annotation.

## 8 Experiments

### 8.1 Vitiligo questionnaires

The seminal study *Vitiligo questionnaire* compiles extensive clinical and demographic data to examine the onset, progression, therapeutic efficacy, and related psychosocial impacts. The dataset is organized into three sheets, classifying patients into three cohorts according to their responses. The questionnaire captures data on demographic information, onset of vitiligo, progression patterns, family history, treatments, and outcomes, offering a comprehensive understanding of the condition. Subsequently, the insights (refer to Table 4) obtained from analyzing the responses across the three cohorts are summarized. Group 1 comprises patients aged 18–40 years, Group 2 includes patients aged 41–60 years, and Group 3 consists of patients aged 61 years and older. The dataset encompasses participants from multiple ethnic backgrounds and Fitzpatrick skin-type categories (I–VI), ensuring representation of varied pigmentation and geographical origins. This diversity was included to mitigate bias in lesion detection performance.

**Table 4:** Summary of vitiligo questionnaire analysis by age group.

**Group**	**Group (18–40)**	**Group (41–60)**	**Age group (61+)**
Demographics	Predominantly urban population with diverse ethnic backgrounds.	Higher proportion of rural respondents compared to younger group.	Majority of respondents are retired and from rural areas.
Vitiligo onset	Early onset (10–20 years) reported in most cases.	Onset primarily between 21–30 years.	Later onset, often reported post-40 years, aligning with other health conditions.
Progression patterns	Rapid progression in the first few years followed by stabilization.	Gradual progression over several years.	Limited progression but higher prevalence of extensive body coverage.
Treatment types	High use of phototherapy and topical treatments.	Combination therapies (systemic and phototherapy) are prevalent.	Lower treatment adherence, with a focus on traditional and complementary medicine.
Treatment outcomes	Moderate effectiveness, with a focus on appearance improvement.	Noticeable improvement in slowing spread; emotional impact remains significant.	Mixed results; some report stabilization, while others experience recurrence.
Psychosocial impact	High levels of emotional distress and self-consciousness reported.	Moderate emotional distress; concerns about social interactions dominate.	Lower emotional distress but higher focus on managing comorbidities.
Family history	Significant genetic links observed, particularly in early-onset cases.	Moderate genetic links; some familial clustering noted.	Low familial association; cases often sporadic.
Research implications	Need for early intervention and targeted therapies for younger demographics.	Importance of tailored treatments addressing social and emotional impacts.	Focus on improving adherence and managing comorbidities in older patients.

This analysis provides a comprehensive evaluation of the questionnaire responses, identifying key patterns and areas for further research or targeted interventions. The *Rosenberg Self-Esteem Scale (RSE)* was used to assess patient self-esteem as a key psychosocial dimension (see Table 5) affected by Vitiligo. The RSE, a reliable and validated instrument, measures self-esteem through 10 items scored on a 4-point Likert scale (1 = Strongly agree, 4 = Strongly disagree). Negatively worded items are reverse-scored to ensure consistency, with higher total scores reflecting lower self-esteem. Data from these groups was analyzed to understand how self-esteem varies across demographic, clinical, and psychosocial factors, enabling insights into the condition's psychological impacts and treatment effectiveness.

**Table 5:** Summary of Rosenberg Self-Esteem scale analysis by age group.

**Group**	**Age group 18–40**	**Age group 41–60**	**Age group 61+**
Self-esteem levels	Moderate to high self-esteem observed; significant variation in cases with visible vitiligo.	Moderate self-esteem, often influenced by social interactions and visible symptoms.	Lower self-esteem levels in cases with comorbidities or extensive skin coverage.
Impact of Vitiligo	Emotional distress linked to self-perception; younger group shows more sensitivity.	Social stigmas significantly impact self-esteem in this group.	Lesser emotional distress but higher focus on managing health and social isolation.
Treatment correlation	Effective treatments improve self-esteem significantly.	Partial improvements in self-esteem noted with slower disease progression.	Treatment adherence challenges result in mixed outcomes for self-esteem.
Research implications	Focus on integrating psychological support with early interventions.	Emphasis on addressing social stigmas and improving treatment access.	Developing holistic strategies to manage comorbidities and emotional wellbeing.

The RSE analysis indicates a significant variation in self-esteem among the groups of patients, with Group 3 (61+) exhibiting the highest levels of emotional distress. These results emphasize the critical need to incorporate psychological support into vitiligo management strategies, particularly for patients at advanced stages of the disease or those demonstrating lower adherence to treatment protocols. Further research could enhance these insights by establishing correlations between self-esteem and various treatment modalities alongside demographic factors. As part of the study, we have utilized a *simplified questionnaire* as well (refer to Table 6). The analysis concentrates on essential indicators, including age distribution, disease onset, visibility of lesions, educational attainment, marital status, and the extent of skin affected. These variables provide a comprehensive examination of the patient demographic and the psychological and social impacts of the disease. This table effectively summarizes the findings, offering a comparative analysis of key variables across the two groups.

**Table 6:** Descriptive comparison of self-esteem scores across patient groups.

**Self-esteem category**	**Group 1 (18–40)**	**Group 2 (41–60)**	**Group 3 (61+)**
Low	~10%	~20%	~35%
Moderate	~25%	~35%	~30%
Moderate to High	~40%	~30%	~20%
High	~25%	~15%	~15%

### 8.2 Clinical signal weighting

We dynamically adjusted the emotional threshold θ_2_ based on the DLQI and RSE scores:


$$\begin{array}{l} \theta_{2}=\theta_{2}^{0}-\lambda_{1}\cdot {\text{DLQI}}_{\text{norm}}-\lambda_{2}\cdot {\text{RSE}}_{\text{norm}} \end{array}$$ (41)

This allows the system to become more sensitive to declines in mental health among already vulnerable patients. The *Dermatology Life Quality Index (DLQI)* questionnaire (2) was used to evaluate the impact of quality of life (QoL) of skin conditions among patients. This validated instrument contains 10 items, each scored on a four-point Likert scale (0–3), yielding a total score range of 0–30. Lower scores indicate minimal impact on life, while higher scores indicate severe QoL impairments. The data set comprises 114 patients. The responses of each patient to the DLQI questionnaire were aggregated into a total score, reflecting their overall impact on quality of life.

## 9 Clinical questionnaire integration

We computed composite scores from DLQI and RSE for each patient. These were correlated with image classifier confidence scores and sentiment polarity.


$$\begin{array}{l} \rho_{DLQI,S_{\text{skin}}}=0.41\;\left(p<0.01\right),\;\rho_{RSE,S_{\text{emo}}}=-0.53\;\left(p<0.001\right) \end{array}$$ (42)

These results show a moderate to strong relationship between the severity of the dermatological image and psychological distress, validating our integrated monitoring hypothesis.

### 9.1 YOLOv11 research

The data set used in this study comprises 3,959 images, evenly distributed between two classes: vitiligo lesions (2,090 images) and healthy skin (1,869 images). Each image is precisely annotated, and the average resolution of 0.41 megapixels (median resolution: 640 × 640 pixels) ensures computational efficiency during training while preserving sufficient detail for accurate detection and segmentation. The balanced class distribution mitigates the risk of model bias, contributing significantly to the high precision and recall values observed during evaluation.

## 10 Results

### 10.1 Vitiligo questionnaires results

Drawing upon the summary of responses delineated in *Vitiligo questionnaire*, a comprehensive analysis reveals discernible patterns across three age cohorts (18–40, 41–60, and 61+ years) in terms of demographics, onset, progression, therapeutic approaches, and psychosocial impacts. The younger cohort (18–40) predominantly resides in urban settings, characterized by early onset and rapid disease progression, necessitating intensive treatment modalities such as phototherapy and topical applications. This group bears a significant psychosocial burden, primarily marked by self-consciousness and emotional distress. Conversely, the middle-aged cohort (41–60) exhibits a demographic mix of rural and urban populations, with onset typically occurring later, between 21 and 30 years. Disease progression within this cohort is more gradual, often managed with combination therapies. Although emotional distress persists, psychosocial dynamics are more strongly influenced by social interactions. The older cohort (61+) typically experiences a later onset, frequently associated with comorbid health conditions, displaying limited progression yet extensive body coverage. This group's adherence to treatment is comparatively lower, with a propensity for traditional or complementary therapeutic approaches. The psychosocial focus transitions from emotional distress to the management of comorbidities. These findings emphasize the necessity for age-specific therapeutic and supportive strategies, with early interventions proving beneficial for the younger groups and comprehensive care addressing comorbidities and adherence necessary for older patients. The evaluation of variations in self-esteem as analyzed in *Rosenberg Self-Esteem (RSE)* underscores the age-related nuances among vitiligo patients. Within the younger age cohort (18–40), self-esteem levels vary from moderate to high, with the visibility of vitiligo exerting a substantial impact on self-perception. Emotional distress is intricately linked to self-perception, highlighting this demographic's heightened sensitivity. Successful treatment modalities have been shown to enhance self-esteem, emphasizing the pivotal role of integrating psychological support with early medical interventions. The middle-aged cohort (41–60) generally experiences moderate self-esteem levels, significantly affected by social interactions and symptom visibility. Social stigma emerges as a predominant factor influencing self-esteem, with treatment-related improvements observed but commensurate with slower disease progression. Addressing social stigma and augmenting treatment accessibility are paramount for this cohort. For the older age group (61+), self-esteem levels tend to be lower, particularly amongst individuals managing comorbidities or widespread skin involvement. While emotional distress is less pronounced, the focus shifts to managing health concerns and social isolation. Adherence to treatment remains problematic, yielding varied self-esteem outcomes. For this group, a holistic approach addressing both comorbidities and emotional wellbeing is crucial for enhancing overall quality of life. These findings underscore the imperative for tailored psychosocial and therapeutic interventions across different age cohorts. A summarized representation of these outcomes is found in Table 7, which elucidates the subtle differences in psychosocial impacts across groups and provides a framework for customized interventions.

**Table 7:** Psychosocial impact comparison across groups.

**Indicator**	**Group (18–40)**	**Group (41–60)**	**Group (61+)**
Visible lesions	0.692	0.526	0.541
No visible lesions	0.308	0.474	0.46
Marital status (married)	0.18	0.868	0.919
Marital status (single)	0.821	0.132	0.081
Skin impact (< 10%)	0.692	0.342	0.378
Skin impact (0%–25%)	0.308	0.447	0.46
Skin impact (25%)	0	0.211	0.162

The *simplified comparison of self-esteem scores between age groups* (see Table 6) reveals distinct patterns influenced by the interaction of age, self-perception, and external factors. In Group 1 (18–40 years), 40% of individuals exhibit moderate to high self-esteem, with another 25% achieving high self-esteem. This reflects the adaptability and responsiveness of the younger population to supportive treatments, despite visible symptoms. Only 10% of this group report low self-esteem, highlighting a relatively positive self-perception. Group 2 (41–60 years) shows a slight decline, with 30% in the moderate to high category and only 15% in the high category. This suggests a greater influence of social stigmas and disease visibility on self-esteem, along with the challenges in maintaining psychological resilience. Moderate self-esteem is more common in this group, 35%, indicating a transitional phase where support systems and treatment outcomes play a crucial role. In Group 3 (61+ years), there is a marked shift, with 35% reporting low self-esteem, the highest among all groups. Moderate and moderate-to-high self-esteem account for 30% and 20%, respectively, while only 15% achieve high self-esteem. This distribution reflects the compounded impact of age-related challenges, such as comorbidities, social isolation, and reduced treatment adherence, on psychological wellbeing. These findings underscore the importance of targeted interventions to boost self-esteem, particularly in older populations, while maintaining support systems across all age groups.

The findings delineated in Table 8 underscore the psychosocial implications of skin conditions in three distinct age brackets (18–40, 41–60, and 61+). Noteworthy observations include the decrement in the prevalence of visible lesions with advancing age as is presented in Table 9. In the youngest cohort (18–40), 69.23% reported visible lesions, whereas this percentage diminishes to 52.63% in the middle-aged cohort (41–60) and 54.05% in the oldest cohort (61+). Conversely, the proportion of individuals without visible lesions augments with age, rising from 30.77% in the youngest cohort to 47.37% in the middle-aged and 45.95% in the oldest cohorts, respectively. Regarding marital status, the proportion of married individuals escalates markedly with age: a mere 17.95% in the youngest cohort are married, contrasted with 86.84% in the middle-aged cohort and 91.89% in the oldest cohort. In contrast, single individuals are more prevalent among the younger demographic (82.05%), with a pronounced decrease in prevalence among the middle-aged (13.16%) and oldest cohorts (8.11%). Skin impact below 10% is most prevalent in the youngest cohort (69.23%) and shows a decline with age, being observed in 34.21% of the middle-aged cohort and 37.84% of the oldest cohort. Skin impact within the range of 0%–25% is more evenly distributed among all cohorts but tends to increase slightly with age: 30.77% in the youngest cohort, 44.74% in the middle-aged cohort, and 45.95% in the oldest cohort. Skin impact surpassing 25% is absent in the youngest cohort yet is observed in older cohorts, with 21.05% of individuals aged 41–60 and 16.22% of those aged 61+. The data imply that visible lesions are more prevalent among younger individuals, while older cohorts exhibit higher rates of marital stability and a broader distribution of skin impact severity. These results highlight potential age-related disparities in psychosocial experiences and coping mechanisms pertaining to skin conditions, thereby suggesting the necessity for age-specific interventions.

**Table 8:** Summary of dermatology life quality index analysis.

**Group**	**Patients**
QoL impact	Moderate to severe impacts were reported by ~45% of respondents, with a mean score of 15.2, while significant quality of life (QoL) impairments were observed in ~60% of respondents, with a mean score of 18.7.
Key insights	Younger patients highlighted impacts on social activities and clothing choices, while older patients focused on physical discomfort and treatment-related challenges.
Research implications	Interventions should be tailored to address lifestyle disruptions commonly experienced by younger populations, while prioritizing the mitigation of physical symptoms and the enhancement of treatment adherence among older cohorts.

**Table 9:** Comparison of key indicators across patient groups.

**Indicator**	**Group (18–40)**	**Group (41–60)**	**Group (61+)**
Age distribution	30–39 years	45–50 years	65–70 years
Disease onset	10–20 years	21–30 years	Post 40 years
Visible lesions	Early and broad lesions in under 5 years	Progressive lesions over time	Late-onset, extensive lesions
Higher education	Common	Less frequent	Least frequent
Married	Less common	More common	Most common
Not married	More common	Less common	Least common
Affected Skin (< 10%)	~50% of cases	~40% of cases	~30% of cases
Affected Skin (>25%)	~20% of cases	~35% of cases	~50% of cases

The Dermatology Life Quality Index (DLQI) scores (see Table 10) offer valuable information on the quality of life impairments associated with dermatological conditions in different age groups. The youngest cohort (ages 18–40) demonstrates the highest mean DLQI score of 15.1795, indicating a moderate level of impairment. The midlife cohort (ages 41–60) has a marginally reduced score of 14, still indicating moderate impairment. In contrast, the senior cohort (age 61 and older) records the lowest mean DLQI score of 10.7568, suggesting only a low level of impairment. Emotional distress manifests as moderate in the younger and middle-aged cohorts but decreases to low levels in the elderly population. This may suggest heightened emotional resilience or acceptance in older individuals. Disturbances in social activity participation are also moderate in younger and middle-aged cohorts, but are perceived as low by older individuals. Younger patients may show increased concern for social appearances, while older individuals might prioritize alternative aspects of life. The effects of dermatological conditions on work or study-related activities are considered moderate among younger patients, but low among middle-aged and senior cohorts, in agreement with the varying intensities of work or study pressures during different stages of life. Treatment-related challenges are consistently rated low in all age cohorts, indicating that issues of adherence to treatment or accessibility may not pose significant barriers. Data indicate that younger patients endure greater psychosocial impacts, attributable to visible lesions, interruptions in social activities, and challenges associated with work or study, as evidenced by their elevated DLQI scores and moderate levels of emotional distress. Middle-aged patients exhibit similar trends, albeit with slightly reduced overall impacts, possibly due to enhanced coping mechanisms developed over time. Seniors experience fewer psychosocial disturbances, characterized by lower DLQI scores and diminished emotional distress, denoting a shift in priorities or increased adaptation to their condition. These observations underscore the need for age-specific interventions, advocating for changes in lifestyle and social concerns for younger patients, while focusing on the management of physical symptoms and the optimization of treatment protocols for older populations.

**Table 10:** DLQI comparison across patient groups.

**Category**	**Group 1 (18–40)**	**Group 2 (41–60)**	**Group 3 (61+)**
Mean DLQI Score	15.18	14	10.76
Emotional distress	Moderate	Moderate	Low
Social activities	Moderate	Moderate	Low
Work/study	Moderate	Low	Low
Treatment issues	Low	Low	Low

### 10.2 YOLOv11 results

**Figure 3:**
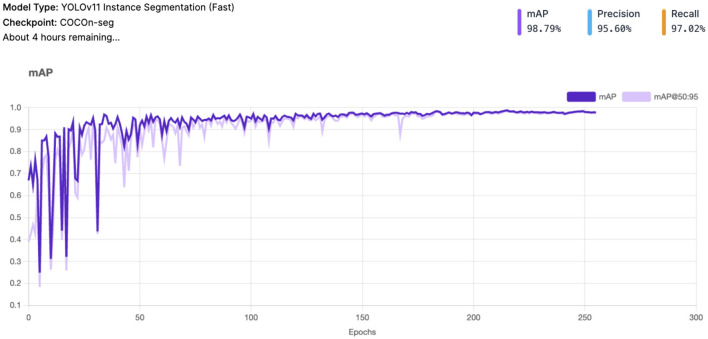
YOLOv11 - mAP and mAP@50:95 result.

The *Mean Average Precision* (mAP) (see Figure 3) achieved a very high value of 98.8%, indicating excellent overall performance in object detection and classification. mAP measures the system's precision-recall balance across different Intersection over Union (IoU) thresholds, making it a comprehensive metric of accuracy.

*Precision* stands at 95.6%, reflecting the system's ability to minimize false positives. This is particularly important in avoiding incorrect detections of vitiligo lesions or healthy skin patches. *Recall* is 97.0%, highlighting the system's robustness in identifying most true positive cases. This ensures that very few Vitiligo lesions or healthy skin patches are missed during detection.

**Figure 4:**
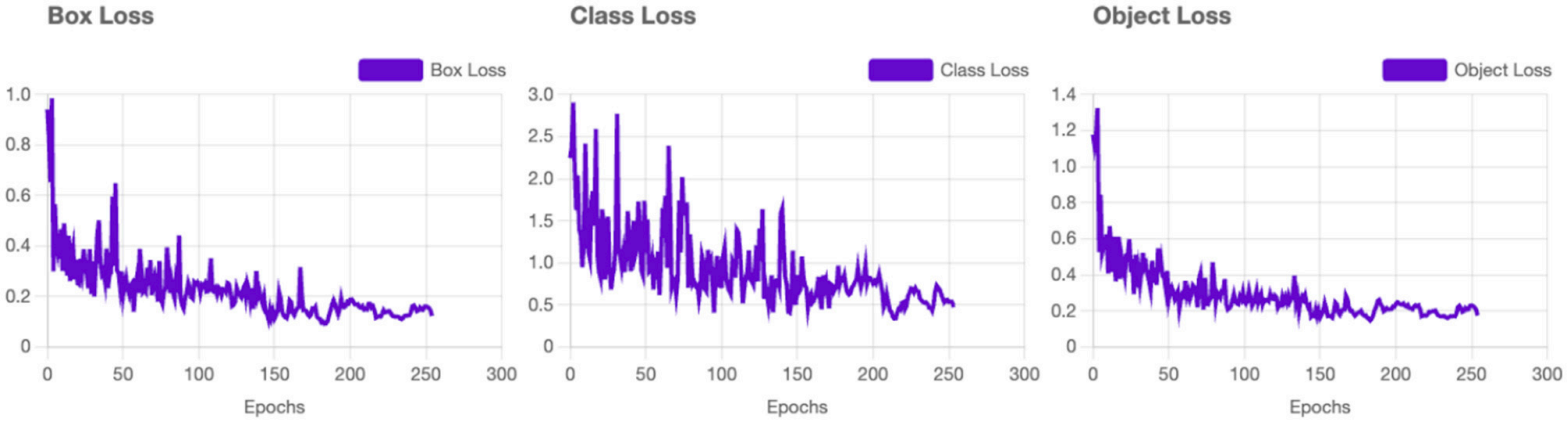
YOLOv11 - Loss results. Training loss and validation loss curves demonstrating model convergence.

*Box Loss* converges smoothly, indicating the model's ability to accurately localize bounding boxes for vitiligo lesions and healthy skin (see Figure 4). The *class loss* shows a gradual reduction which confirms the system's ability to distinguish between vitiligo and healthy skin effectively. Distribution Focal Loss (*DF Loss*) shows stable decline demonstrates effective regression of the shapes and sizes of the bounding box. The *Segmentation Loss* shows a rapid convergence with minimal oscillations suggesting the system is accurately segmenting areas of interest (e.g., vitiligo patches).

**Figure 5:**
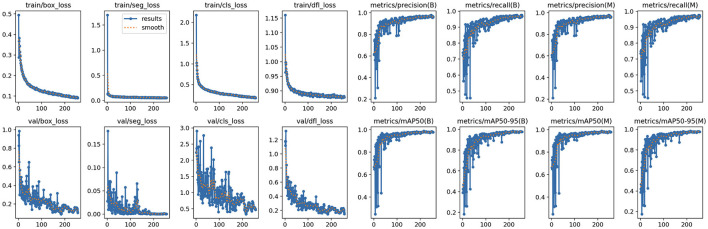
YOLOv11 - Training graphs. Training loss and validation loss curves demonstrating model convergence.

*Validation loss* curves are consistent with the training loss curves (see Figure 5), which confirms that the model generalizes well without overfitting. Minor oscillations are expected due to the complexity of the dataset. *Precision and recall metrics* improve steadily and plateau near their final values, reflecting strong model learning dynamics. The high precision-recall balance is indicative of minimal trade-offs between the two metrics. The *mAP and mAP@50:95* both stabilize after the initial epochs, reaching values close to 1. This confirms that the model is not only precise but also consistent between varying IoU thresholds.

### 10.3 NLP component results

The classifier achieved an F1 score of 0.83 on the validation set. Table 11 shows the performance metrics.

**Table 11:** Sentiment classifier performance on GoEmotions subset.

**Model**	**Accuracy**	**F1-Score**	**AUC**
BERT-base (finetuned)	0.82	0.79	0.87
Naive Bayes baseline	0.62	0.58	0.66

## 11 Discussion

**Figure 6:**
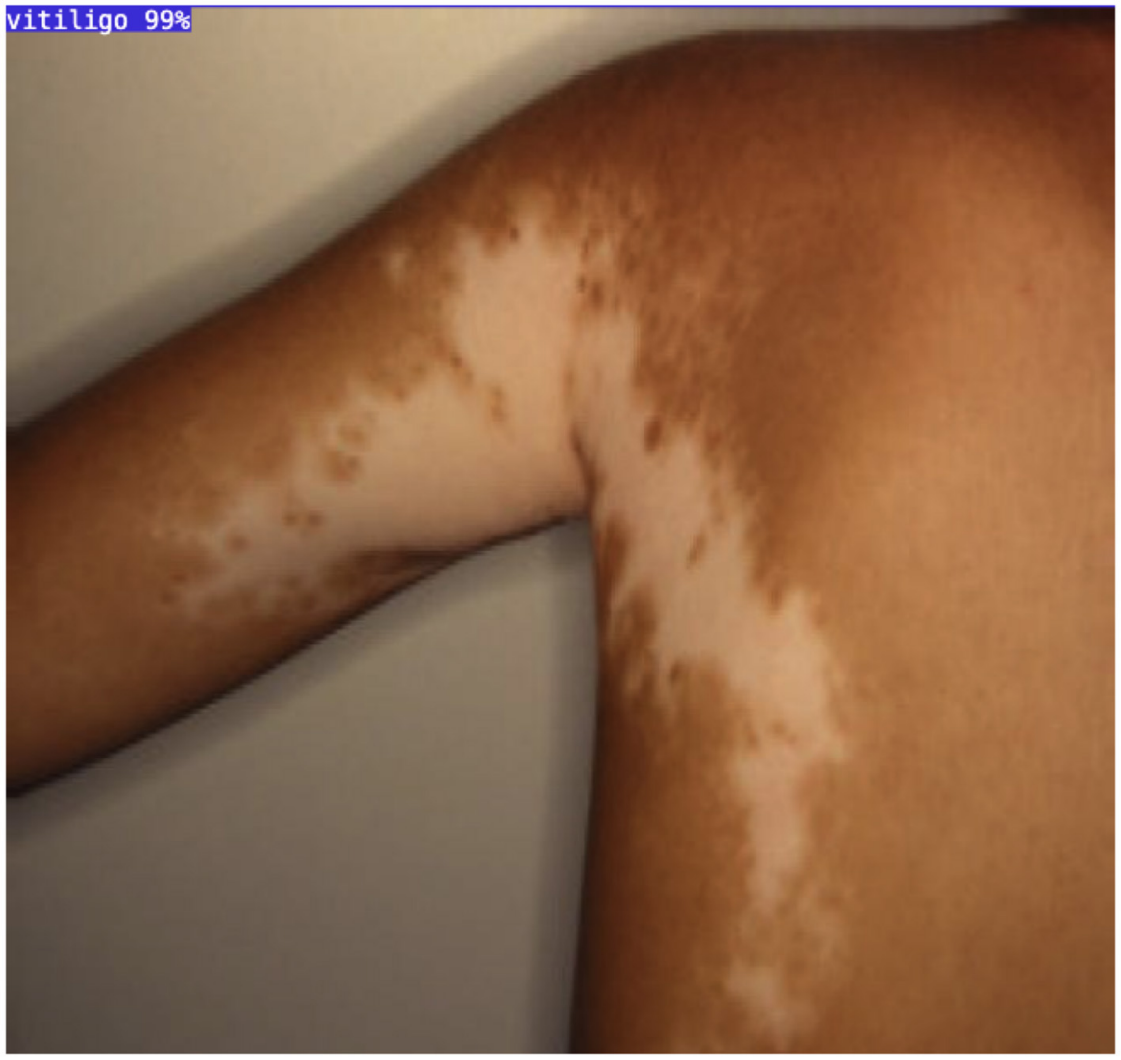
YOLOv11 detection output showing vitiligo lesions in a smartphone image.

The proposed AI-driven framework for Vitiligo detection and classification demonstrates exceptional performance in all key evaluation metrics, establishing its potential as a robust diagnostic tool in dermatology (Figure 6). With a *mean average precision* (mAP) of 98.8%, *Precision* of 95.6%, and *Recall* of 97.0%, the system maintains an optimal balance by reducing false positives while effectively identifying most true positives. This balance is critical to ensure reliable diagnostic results, particularly in clinical settings. The loss curves for training and validation (including box loss, classification loss, segmentation loss, and distribution focal loss) exhibit smooth convergence, with minimal oscillations. This reflects effective model learning and generalization to unseen validation data. The parallel trends between training and validation losses indicate that the model avoids overfitting, even with the complexity of the dataset. Throughout the training process, both precision and recall metrics exhibit a steady increase, eventually stabilizing at elevated values. A precision of 95.6% emphasizes the model's ability to reduce the occurrence of false positives, ensuring that healthy skin is not erroneously labeled as vitiligo. At the same time, the recall of 97.0% illustrates the ability of the model to identify almost all vitiligo cases, thus reducing the chances of overlooked diagnoses. The mAP of 98.8%, coupled with stable performance across the IoU thresholds (mAP @ 50:95), indicates exceptional precision in detecting and classifying lesions. This high mAP ensures that the model maintains precision and recall across varying overlaps of the boundaries, *that ensures its applicability in practical scenarios*.

### 11.1 Comparison with prior work

Unlike earlier studies that addressed dermatological imaging and mental health in isolation, our framework unifies the two domains. Previous vitiligo studies focused primarily on clinical dermatology or psychosocial surveys, without multimodal integration. Our system represents, to our knowledge, the first AI prototype linking YOLO-based skin analysis with NLP-driven emotional monitoring. Future work will integrate patient-reported messages for sentiment training, validate the multimodal fusion in clinical cohorts, and extend the framework to other chronic dermatological conditions with strong psychosocial components. A prospective clinical validation study is planned to evaluate the fusion alert mechanism in real-world workflows, assessing its impact on patient outcomes and clinician response time.

### 11.2 Clinical implications

Integration of dermatological and psychosocial monitoring provides physicians with a decision support tool for holistic patient care. Mobile deployment improves accessibility in low-resource settings, while continuous sentiment monitoring fosters proactive interventions. The high sensitivity and specificity of the system have significant clinical implications. With high recall, the system ensures that vitiligo lesions are reliably identified, supporting early diagnosis and timely intervention. Simultaneously, high precision reduces false positives, preventing unnecessary clinical evaluations for healthy individuals. This dual capability improves the reliability of the system, ensuring both efficiency and trustworthiness in practical deployment. The resolution of 640 × 640 pixels provides an ideal compromise between computational efficiency and diagnostic precision, making the system highly suitable for scalable use in settings with limited resources, such as mobile or edge healthcare solutions. Additionally, the well-balanced dataset and accurate annotations enhance the model's capability to generalize across a wide variety of patient scenarios.

### 11.3 Limitations

The sentiment classifier was trained on public data (GoEmotions) rather than patient-reported messages, which may limit clinical generalizability. Fusion experiments were simulated; real-world validation through prospective trials is necessary. Future work will integrate in-app patient data and extend the model to other dermatological conditions. The sentiment classifier, trained on the GoEmotions corpus, serves as a transferable baseline; Future clinical validation will employ patient-generated text and voice inputs from vitiligo cohorts to fine-tune and benchmark real-world performance.

### 11.4 AI-driven ethical considerations

Due to the nature of the disease and its effect on other fields such as mental health or phycology, the adoption of AI-driven technologies in healthcare, especially in the management of sensitive conditions such as Vitiligo, requires a rigorous ethical framework to ensure the responsible use of technology. Addressing ethical considerations is critical to safeguarding patient rights, building trust, and maximizing the societal benefits of this innovation.

1. *Algorithmic bias and fairness* also warrant careful attention (30). AI models can unintentionally reflect biases present in training datasets, leading to disparities in system performance between demographic groups, such as those differentiated by skin tone, gender, or language. To mitigate such risks, training datasets must be diverse and representative. Furthermore, the system must be designed to ensure equitable access, particularly in low-resource or underserved settings, to prevent exacerbating existing disparities in healthcare.
2. The *psychological impact* of system interactions is another critical consideration (4). Vitiligo often carries a significant emotional burden, and the AI system must be designed to respond with empathy and sensitivity. Responses must be carefully calibrated to avoid reinforcing negative self-perceptions or causing additional distress. Trigger management mechanisms should also ensure that incorrect or poorly contextualized responses do not adversely affect patients mental health.
3. *Accountability* and human oversight are fundamental to maintaining ethical standards (31). While the AI system provides diagnostic and monitoring support, it must not replace human judgment. Clinicians must remain central to the decision-making process, ensuring that interventions or treatments are guided by professional expertise.
4. *Transparency and explainability* are crucial to building trust in AI technologies (32). Patients and clinicians should have access to clear explanations of how the AI system generates its output. This transparency fosters confidence in the technology while allowing users to understand the basis of its recommendations. The design of algorithms must prioritize interpretability, ensuring that decisions are not perceived as “black boxes.”
5. *Compliance with regulatory frameworks* that govern medical devices and AI systems is imperative (33). Research must comply with relevant local, national and international regulations, and ethical approvals from Institutional Review Boards (IRBs) or ethics committees must be obtained before conducting the study.
6. The *sustainability* and *monitoring of the AI system* are equally important (34). Regular post-deployment evaluations should assess its ongoing *ethical impact*, with patient feedback incorporated to refine and improve the system. In addition, researchers must address dual use risks by proactively establishing guidelines to prevent misuse of technology, such as unauthorized emotional profiling or data exploitation. Continuous user-experience monitoring will be embedded to evaluate and mitigate any unintended psychological impact, ensuring that AI-generated feedback remains supportive and non-intrusive.

In conclusion, in projects such as the one presented in this article, addressing these ethical considerations ensures that this research respects patient rights, promotes equity, and improves the legitimacy of AI-driven healthcare solutions. Integrating ethical principles into the system's development and implementation holds the potential to enhance patient care and foster long-term societal trust in AI technologies.

### 11.5 Limitations of the approach

Although the proposed AI-driven framework for Vitiligo diagnosis and mental health monitoring has significant potential, it is not without limitations: (1) *Bias and fairness* (35): algorithmic bias remains a critical concern. Imbalances (36) in training data sets -such as underrepresentation of certain skin types, languages, or age groups - can result in disparities in model performance. This could lead to unequal diagnostic results or misinterpretation of emotional states, disproportionately affecting vulnerable populations; (2) *Complexity of emotional analysis* (37): mental health assessment through sentiment and emotion analysis involves subjective interpretations, which may not always align with clinical evaluations. Emotional states are nuanced and influenced by context, personal history, and cultural factors, which can complicate accurate detection and response generation; (3) *Integration of multimodal data* (38): While integrating text and audio inputs can improve system precision, it also increases complexity. The fusion of multimodal data requires sophisticated models and computational resources, which can limit real-time processing and accessibility, particularly in resource-constrained environments. To manage this complexity, modular processing and lightweight model compression strategies are being explored to preserve real-time responsiveness on mobile devices; (4) *Technical challenges in speech-to-text* (39): Speech-to-text technology, while advanced, is not immune to errors, especially in cases of diverse accents, dialects, or background noise. These inaccuracies could negatively impact the analysis of downstream emotions and sentiments, leading to incorrect conclusions or inappropriate interventions; (5) *Dependency on technology* (40): reliance on AI systems may inadvertently reduce human oversight, leading to a potential overreliance on automated decisions. Without proper checks, this could result in missed opportunities for nuanced clinical judgments that require human intuition and experience; (6) *Acceptance and trust* (41): adoption of AI-driven healthcare tools requires a high level of trust from both patients and clinicians. Concerns about the interpretability and reliability of the system may hinder its acceptance, particularly in settings where human-centric care is deeply valued; (7) *Real-time adaptation* (42): While reinforcement learning and feedback loops are incorporated to dynamically improve the system, real-time adaptation to diverse patient inputs and contexts remains a complex task. The system may struggle to balance responsiveness with consistency, particularly in unpredictable scenarios.

Addressing these limitations will require continuous refinement of the models, careful ethical oversight, and collaboration between AI developers, clinicians, and policy makers. By recognizing these challenges, research can prioritize strategies to mitigate them, ensuring that the proposed framework achieves its full potential to improve Vitiligo care and mental health support. Future work could also explore the integration of advanced AI techniques, such as reinforcement learning and Retrieval-Augmented Generation (RAG), to optimize real-time decision making and patient interaction.

Although the current study implements the dermatological AI pipeline, sentiment- and speech-based monitoring is still under development. No large-scale clinical validation has been performed. Future work includes testing the system with real patient interviews, mobile deployment, and longitudinal tracking.

## 12 Future implementation lines

*Retrieval-Augmented Generation* (RAG) combines neural networks with information retrieval systems to dynamically generate responses grounded in external knowledge (43). Potential applications include: (1) *Knowledge-driven support* provides patients with context-specific educational materials and coping strategies. (2) *Clinician assistance* retrieve clinical guidelines and case studies to support decision-making; (3) *Speech-to-Text Integration* could generate empathetic and context-sensitive responses based on the patient's history. By leveraging *Transfer learning*, it can be accelerated the development of AI models and ensure adaptability with: (1) *domain adaptation*, tuning pre-trained models (e.g., BERT, GPT) for healthcare-related purposes like sentiment analysis and detecting Vitiligo; (2) *Cross-language support*, which will adapt multilingual models for diverse populations; the (3) *Low-resource scalability*, which could train models effectively in environments with limited labeled data. By integrating *reinforcement learning* (RL), enables systems to adapt and improve through feedback: (1) *Personalized interventions*, which will optimize responses based on prior patient interactions; (2) *Adaptive feedback*, which could dynamically adjust the feedback mechanisms to guide patients toward acceptance; the (3) *real-time learning*, which continuously will refine the applied strategies as new data becomes available. Another promising line would be the *Multimodal and scalable implementation* of proposed method, where with the (1) *Multimodal integration* it is possible to combine the text, audio, and wearable device data for holistic analysis; (2) with a *Telemedicine deployment* it is possible expanding access to underserved areas through scalable, cloud-based solutions; (3) with an integration of *population health insights*, the system could aggregate the anonymized data to identify trends in Vitiligo care and mental health. Another aspect would be the implementation of *Ethical and transparent AI*, which might have the following line of research: (1) *Explainable AI (XAI)* (32), to develop transparent models to foster patient and clinician trust; the (2) *Bias mitigation* line, to ensure the equitable performance across diverse demographic groups; and the (3) *Regulatory compliance* line, to adapt to evolving healthcare the new AI standards. The impact of AI-supported technologies: (1) *RAG* enables evidence-based, real-time responses by integrating external knowledge; (2) *Transfer Learning* reduces development time and enhances adaptability to new contexts; (3) *Reinforcement Learning* optimizes interventions through continuous feedback and personalized strategies. This component remains a planned extension of the system. Future versions will include Whisper-based transcription pipelines combined with prosodic analysis for emotion detection from voice.

## 13 Conclusions

Our prototype represents a foundational step toward a fully integrated AI framework for dermatology and mental health. Future work includes longitudinal patient validation and transfer learning from larger multimodal corpora. This study investigated the incorporation of cutting-edge AI technologies to tackle both the physical and psychological aspects of Vitiligo treatment and physiological and/or mental monitoring. Using state-of-the-art approaches such as the YOLO algorithm (v11 version) for real-time and *accurate Vitiligo detection* and *sentence analysis* for *continuous mental health monitoring*, the framework presents a holistic, patient-centered solution. The findings and proposed methodologies align with the objectives outlined at the start of the study, demonstrating significant potential to improve clinical outcomes and patient quality of life. Even in diverse and resource-limited environments, YOLO improves the diagnosis of Vitiligo lesion. Dermatologists can quickly diagnose and treat patients with real-time functionality, a key care gap. With sentiment analysis and speech-to-text, the system monitors patients' emotional health beyond medical care. Real-time emotional analysis identifies mental health problems early, enabling proactive treatment and acceptance. Lightweight AI models on mobile and cloud platforms can improve diagnosis and monitoring in disadvantaged areas, according to the proposed strategy. It satisfies the global needs for equity in healthcare. Holistic care addresses dermatological and psychological requirements. The paradigm breaks the boundary between physical and mental health management, allowing doctors to provide more personalized and compassionate care. This study shows how artificial intelligence and healthcare can help dermatologists, mental health professionals, and technology work together. Health problems are complex and require interdisciplinary approaches. Scalable and flexible AI solutions use RAG, transfer learning, and reinforcement learning. These technologies improve healthcare care by improving diagnosis, patient participation, and focused interventions. This study greatly affects healthcare care. AI improves vitiligo treatment and supports other chronic psychological disorders. The system addresses the goal of equitable and sustainable healthcare through accessibility, ethics, and real-time adaptation. Data quality, algorithmic bias, and scalability in resource-limited contexts must be examined, although the proposed solution fits multiple needs. Future research should emphasize multimodal integration, AI-driven assessment transparency, and cross-cultural and linguistic applicability. Modern methods like RAG and reinforcement learning may help overcome these limits. AI in healthcare could change Vitiligo treatment, according to this study. Research addresses physical and emotional health and advances AI-assisted medicine in a patient-centered manner. The proposed approach could revolutionize the treatment of chronic diseases worldwide by improving patient outcomes through continuous improvement and collaboration. With exceptional mAP (98. 8%), precision (95. 6%), and recall (97. 0%), the system demonstrates robust diagnostic capabilities, supported by consistent convergence of loss curves and strong generalization to validation data. These findings underscore the suitability of the framework for clinical applications, offering a scalable and efficient solution for early vitiligo diagnosis. By tackling noted constraints and broadening its framework to encompass comprehensive patient monitoring, this paradigm could significantly alter the standard of care in dermatology. The integration of dermatological AI with emotional monitoring opens a new frontier in patient-centered, multimodal digital health for chronic conditions like vitiligo.

## Data Availability

The original contributions presented in the study are included in the article, further inquiries can be directed to the corresponding author.
